# Mandatory or voluntary labeling of genetically modified foods: Consequences for the Canadian canola sector

**DOI:** 10.1080/21645698.2026.2703716

**Published:** 2026-08-18

**Authors:** Stavroula Malla, Kurt Klein, David Forestell

**Affiliations:** Economics Department, University of Lethbridge, Lethbridge, Alberta, Canada

**Keywords:** Canola sector, economic policies and regulations, mandatory labeling, voluntary labeling

## Abstract

A bioengineered food labeling law recently came into full effect in the U.S. making the U.S. the 65th country with a mandatory GM (genetically modified) labeling policy. In contrast, there is no mandatory labeling of GM foods in Canada, including the canola industry’s food products‚ such as canola oil and meal, despite intensive public campaigning and 20 years of polling that consistently show that more than 80% of Canadians want these products labeled. This paper examines the economic impact of the new U.S.’ and other countries' laws regarding mandatory labeling of GM food products on the Canadian canola sector‚ overall economy, and society. It also considers the case for mandatory labeling of GM food products in Canada. Policy implications are discussed, and several policy recommendations are made that could contribute to the canola sector’s growth, potential improvements in individual wellbeing, and increased overall social welfare. This paper sheds light on the important but neglected issue of GM food labeling policy in Canada and its potential implications for the Canadian canola industry.

## Introduction

New labeling requirements for genetically modified (GM) foods in the U.S. might pose significant challenges for exports of Canadian agricultural and food products; the U.S. law uses the term bioengineered (BE) rather than GM. The U.S. is the destination for more than 85% of all Canadian canola meal and up to 60% of total canola oil exports.^1^ Almost all canola grown in Canada is from genetically modified stock. Imposing labeling regulations that differ across borders can create significant trade barriers and additional costs for producers.

In Canada, labeling of GM foods, including canola sector’s food products, is voluntary unless the resulting product has a significant change in the nutritional or compositional structure that poses a health or safety concern to consumers (e.g., change in allergens), in which case labeling of both GM products and non-GM products is mandatory (e.g.,^2–4^). Both Health Canada and the Canadian Food Inspection Agency (CFIA) jointly govern mandatory and voluntary food labeling regulations to ensure that the products meet safety standards and that labels are factual and not misleading.

In July 2016, the U.S. amended the Agricultural Marketing Act of 1946 to include the National Bioengineered Food Disclosure Standard, which legislates the mandatory labeling of GM foods.^5^ Prior to this date, there was no requirement for GM labeling (as remains the case in Canada). Additionally, in the U.S., labeling of GM products was required only if the food product was compositionally different than its original counterpart.^6,7^ For example, a canola oil with elevated levels of lauric acid had to be labeled as “laurate canola oil.” Under the amended law, GM foods are required to have either a label with a disclosure statement, a symbol, or an electronic digital disclosure.^5^ Following a phase-in period, the U.S. government “National Bioengineered Food Disclosure Standard” has been in full effect since January 2022.

As a result of the new regulation, sellers of GM food products in the U.S. market face an increasing need for identity preservation and segregation systems in addition to ensuring products meet the new U.S. labeling requirements. However, the inability and a lack of technology to effectively segregate GM crops from non-GM crops are widely recognized as a significant economic challenge and a costly issue for producers (e.g.,^8–12^). Not only is segregation now a concern for trade with the U.S., but it also remains an issue for trade in other global markets due to international differences in policies that regulate GM crops. Currently, 65 countries around the world require labeling of genetically modified foods, including most European countries, China, Russia, Japan, Brazil, and South Africa. These requirements are voluntary restrictive practices that are consistent with initial WTO’s regulations on GM products and the Codex Alimentarius^13,14^)

A growing body of literature has examined the economic and consumer implications of GM labeling policies in the U.S. but the economic consequences for the Canadian canola sector have not been assessed in any academic literature (e.g.,^15–25^). A goal of this paper is to acquire a better understanding of the economic, social, and regulatory issues surrounding the GM labeling scheme. Specifically, the objective of the paper is to assess the economic consequences of the new U.S. (PL 114–216) and other countries’ regulations of mandatory GM food labeling on the Canadian canola sector. It also evaluates the impact on agricultural trade, international market access, and competitiveness of Canadian canola products in the global agricultural economy. Further, the paper examines the case for a new regulation that would impose mandatory labeling on GM food products in Canada, including canola products, and assesses its potential economic consequences, i.e., the expected benefits and costs on the Canadian canola industry.

This article begins by reviewing aspects surrounding GM labeling issues, including an overview of the GM canola industry in Canada, the Canadian government’s role, the role of nongovernmental organizations (NGOs), and the Canadian consumer’s relationship with labeling. It then assesses the potential costs and benefits of mandatory labeling while making a distinction between existing Canadian canola sector trade relations and the creation of new trade relations within the Canadian canola sector. The article then provides an overall assessment of the study’s findings and concludes by considering various policy implications and recommendations.

The results of this study are potentially of interest to a wide range of academics, industry players, and policymakers in all levels of government because the economic consequences of the new U.S. and other countries’ GM food labeling regulations for the Canadian canola sector, as well as the benefits and costs associated with mandatory labeling of GM canola in Canada have not been assessed in the academic literature.

## Analytical Framework and Research Questions

Public policy debates surrounding food labeling and biotechnology regulation are shaped by the interaction of scientific risk assessment, consumer information demands, advocacy activity, and economic considerations. To analyze these dynamics, this study draws on a policy framework that integrates insights from the literature on regulatory governance, consumer information policy, and political economy.

Consumer information policy introduces an additional dimension to this regulatory landscape. Labeling requirements often are justified based on consumer “right-to-know” arguments, which emphasize transparency and informed choice within food markets (e.g.,^20,25–27^). However, economic research suggests that labeling policies might also generate supply chain costs associated with product segregation, traceability systems, and regulatory compliance (e.g.,^16–22,28,29^). These costs might ultimately be transmitted to consumers through higher food prices.

Finally, policy debates over biotechnology regulation frequently involve the interaction of multiple stakeholders, including government regulators, industry actors, and civil society organizations. These groups differ substantially in their interpretation of scientific evidence, perceptions of technological risk, and policy preferences regarding consumer information.

Taken together, this framework highlights the importance of examining biotechnology labeling policy as the outcome of interactions among regulatory institutions, economic incentives, public perceptions, and advocacy dynamics. This perspective guides the analysis presented in the following sections of the paper.

Overall, this paper examines the potential implications of mandatory GM food labeling in Canada within the context of the country’s existing biotechnology regulatory framework. Specifically, the analysis addresses three research questions:

1. How does Canada’s biotechnology regulatory system shape the policy debate surrounding GM food labeling?
2. What role do advocacy organizations and public opinions play in influencing this policy discussion?
3. What are the potential economic implications of introducing mandatory labeling requirements for Canadian consumers?

By examining these issues, this paper contributes to the broader literature on biotechnology governance, consumer information policy, and food regulation. Understanding how regulatory institutions, advocacy dynamics, and economic considerations interact is critical for evaluating policy proposals related to mandatory GM food labeling in Canada.

## Aspects and Overview Surrounding GMO Labeling Issues

Despite overwhelming scientific evidence to the contrary, there exists a persistent belief that GM food products pose a substantial threat to human health and safety of the environment (e.g.,^30,31^). As consumers cannot directly verify GM attributes through consumption, demand is shaped by knowledge, fear, and trust rather than price or product characteristics. Knowledge has been recognized as a way of reducing affective fear and thereby alter consumer preference formation. For labeling policy, this implies that labels alone might not efficiently correct information asymmetry unless paired with explanatory information capable of changing perceived risk.^32^ To determine whether this is a substantive claim, the concept of “substantial equivalence” was developed by cooperating international authorities to address and evaluate crops produced through biotechnology modification methods.^1^1“Substantial equivalence” is the concept whereby the safety of a new food, particularly one that has been genetically modified (GM), can accurately be assessed by comparing it with a similar “traditional” food that has proven safe in normal use over time.^112^

Specifically, the Organization for Economic Co-operation and Development (OECD), World Health Organization (WHO), and Food and Agriculture Organization of the United Nations (FAO), under the Codex Alimentarius Commission,^14^ agreed that if any GM crop food composition is the same, or similar, to that of a non-modified crop that has a history of safe use for feed and food, except for the expressly modified differences, the GM crop is considered “as safe as” the nonmodified varieties.^33^ In Canada, 99.5% of all canola grown is a GM cultivar.^34^ The aforementioned world authoritative bodies have indicated that this type of canola is safe for feed and food.

### Canada’s GM Canola Sector: An Overview

The agricultural industry is considered one of the main drivers of the Canadian economy.^34^ Canola has become a dominant crop in Canada, primarily due to the introduction of modern biotechnology (e.g.,^35–37^). Over the last few decades, the Canadian canola sector has undergone a transformation from a minor to a major and dominant crop of prairies. The sector has experienced significant growth, including an increase in the area seeded to canola cultivars, the number of canola cultivars available, and yields that have been on an upward trend. Specifically, since the introduction of the first genetically engineered canola seeds in 1995, yields have been steadily increasing, nearly doubling production from 21.8 bushel/acre to 41.8 over a 25-year span with a relatively steady rate of growth. From 1986 to 2016, the total area under canola production increased 313% (6.5–20.8 million acres).^38^ Furthermore, the redistribution of multi-crop farms to canola-predominant farms shifted from being the seventh-largest field crop by area in 1981 to the largest field crop by 2016. Since 2002, GM canola has been present throughout all Canadian growing regions.^39^

In the mid-1990s, Canada began growing commercial GM crops that incorporated many of the cultivar improvements developed by Canadian researchers and biotech teams. Qualitative indicators, levels of desirable fats and proteins, steadily increased with consistently reproducible results (CCC, 2024e). These crops used genetically engineered seed stock to produce a harvest with customized traits. These traits were achieved through manipulation of the desired agri-product’s genetic sequence using accelerated processes to introduce, enhance or delete specific genes. This modification differs from the evolution of a specific crop species, whether through naturally occurring genetic mutations or traditional crossbreeding, due to the ability of advanced engineering techniques to introduce cross-species or cross-kingdom traits.^40^

GM canola accounts for 99.5% of all canola grown in Canada and is now considered the default choice for agri-industry production.^39^ Through the application of GM scientific techniques such as Clustered Regularly Interspaced Short Palindromic Repeats (CRISPR) or the Technological Agnostic Long-Read Analysis Pipeline for Transcriptome Discovery and Quantification (TALON), the rapid development of food crops has become possible. A naturally occurring bacteria or gene is inserted into the organism being modified.^41^ As there is no material difference between the progenitor of the gene and the “new” organism, there can be no differentiation between the two organisms or subsequently derived products. Simply put, if the parent plant is desirable, the offspring must also be. This precedent was established by the FDA in 2001.^42^ Unlike animal husbandry, the ability to produce evidentiary results does not rely on prolonged gestation periods or maturing processes, allowing oilseed and cereal crops to benefit from increased levels of R&D. The “potentiality” of the hazard resulting from the method by which GMOs are produced remains inconclusive.^43^

Canola, as a high-input industrial seed crop, demands intensive resource management, particularly in fertilizer application, where nitrogen, phosphorus, potash, and sulfur must be precisely timed and measured to optimize yields while minimizing environmental harm (e.g.,^44^). The use of large-scale, diesel-powered farm equipment in expanding canola area has raised concerns about CO_2_ emissions; however, precision agriculture technologies and low-tillage practices enabled by GM canola have led to significant emission reductions–estimated at 216 million kg of CO_2_ in 2018—while also improving soil carbon retention (e.g.,^45^). Additionally, the water system impacts of canola production have been mitigated through GM traits that allow for localized herbicide application and enhanced drought tolerance, thereby reducing chemical runoff and irrigation needs (e.g.,^46–50^). Thus, the concern that GM canola contributes more to environmental degradation than “traditional varieties” is shown to be less than factual.

Furthermore, while first-generation biotech crops focused on improving agronomic traits to benefit farmers, second-generation agricultural biotechnology research has focused more on improving the “functional” attributes or nutritional quality of crops (e.g.,^36,51,52^). Second-generation GM crops present an opportunity to address public health issues related to nutritional deficiencies and rising healthcare costs. For example, there have been identified nontrivial direct and indirect health benefits or healthcare savings associated with canola varieties that have improved oil profiles with no trans-fat, low saturated fatty acids and increases in monounsaturated and polyunsaturated fatty acids that have associated health benefits from reduction in cholesterol and reduced risk of cardiovascular diseases (e.g.,^35,53–58^^59–62^).

The process by which canola and other GM crops have been tested in Canada is robust. A combination of growing conditions and environmental ranges to ensure a significantly varied outcome, broad samples of non-modified varieties against which to test extant traits, and statistical modeling resulting in intervals derived from cumulative historical composition data are all factors used to measure the performance and safety of GM crops^63,64^). As GM crops are not always intended for direct human consumption, but rather as feed for livestock, extensive tests have also been conducted to verify their safety for livestock, and by extension, for human consumption. Additionally, subchronic rodent feeding tests have been conducted utilizing both the control and experimental groups, with the latter group receiving up to 40% of their entire nutritional profile from GM crops. Together, the livestock and rodent studies represent a “hybrid of nutritional performance,” providing demonstrable proof that the technological system used to create GM crops is not inherently dangerous to consumers.^33^

Hence, canola is a significant crop for the Canadian economy, and the newly developed GM cultivars of canola are safe, effective, and efficient, with substantial benefits to producers, consumers, and the environment.

### Canadian Government’s Role

In Canada, GM crops (including canola) are subjected to a regulatory process that involves creating a new system of classification: plants with novel traits (PNTs)^2^2An important concept in food labeling policy is the distinction between product-risk-based regulation and process-based regulation. Product-risk-based regulatory systems evaluate products according to measurable safety or environmental risks, regardless of the production method used. In contrast, process-based regulatory approaches trigger regulatory oversight based on the technology used to produce the food product. Canada’s biotechnology regulatory framework reflects a predominantly product-risk-based approach through the regulation of Plants with Novel Traits (PNTs), whereas jurisdictions such as the European Union have historically adopted more process-oriented approaches to biotechnology governance.Canada has adopted a science-based regulatory system centered on the concept of Plants with Novel Traits (PNTs). Under this framework, regulatory oversight is triggered by the presence of novel characteristics rather than using genetic engineering as a breeding method. As a result, genetically modified crops are evaluated under the same risk-assessment framework as other novel agricultural products.,^3^3Recent regulatory guidance has clarified the treatment of gene-edited crops within Canada’s Plants with Novel Traits (PNTs) framework. In 2022, Health Canada indicated that gene-edited plants that do not contain foreign DNA and do not introduce novel risks may be treated similarly to conventional varieties^3^ The Canadian Food Inspection Agency issued similar guidance in 2023, and in 2024 the CFIA Feeds Division confirmed comparable treatment for livestock feed derived from such crops.^65,67^ PNTs are plants that do not have a history of use in Canada or are engineered plants created using transgenic or mutagenic methods with traits that exceed the “normal” range of expression for that specific trait. This classification system, last updated in 2012, remains in use in Canada and is responsible for assessing the safety of new plant species for both the Canadian public and the environment.^65^

Some countries, such as the U.S. historically and Canada, implemented a voluntary GM labeling scheme^4,42^. The main argument for using a voluntary labeling scheme is that the market offers the appropriate labeling incentives and produces an optimal degree of segregation among products without the unnecessary costs a mandatory scheme implies.^15^ Other countries, such as the European Union member states, Australia, New Zealand, Japan, and recently, the U.S., implemented a mandatory GM labeling scheme best served consumers’ “right to know”^5,18,66^.

This “right to know” is established and upheld in Canada by the federal government and is facilitated directly by the Office of Consumer Affairs (OCA). In conjunction with the federal government’s commitment to transparency, the policy of “open data, open information and open dialogue,” is employed and the OCA oversees the legislation administered by the CFIA.^67^ This agency is tasked with providing standards, in conjunction with the Canadian Food and Drugs Act, that establish food labeling policies with respect to health and safety. Regarding GM products, CFIA can mandate specific label requirements where the health and safety of consumers or the environment might be affected.^34^

While voluntary labeling is permitted in Canada, the labels must not relate to the ultimate safety of the product. To facilitate the greater adoption of voluntary labeling, the federal government has been involved in developing national standards to provide guidance and promote the wider adoption of labeling practices. In 2021, the Standards Council of Canada (SCC) officially adopted the recommendation of the Canadian General Standards Board (CGSB), first established in 2004, regarding the advertising and voluntary labeling of foods that are, and are not, products of genetic engineering.^4^

According to Bain and Dandachi,^68^ there are obvious shortcomings to promoting voluntary labeling. The inconsistent application of these labels and the non-enforceability of such initiatives have few guarantees of directly informing the Canadian public of the CFIA’s specific concerns illustrate the problem of voluntary labeling. The additional burden of verifying claims of potential hazards to both the environment and consumers is particularly onerous and costly. However, voluntary labels have the potential to quell the GM labeling debate by placating the desires of consumers who desire greater choice and transparency while simultaneously diminishing agri-food industry concerns about mandatory labeling. With GM crops likely to continue to expand as a mainstay of Canada’s agri-food industry, voluntary labels are unlikely to fundamentally address the social concerns of the anti-GM movement.

Canada’s process of approval and adoption is consistent in its standards and uses the combined forces of Health Canada and the CFIA to create its own food safety framework.^69^ The canola that Canada produces is primarily intended for the international market and is subject to further inspection and regulation by importing countries.

International commodity trade has never taken place at a purity level of 100%. This is particularly challenging, as the purity level for certified seed–the seed that the pedigree seed industry sells to farmers–is typically 99.5% pure. If Canadian producers wish to export to these countries, they must provide appropriate information regarding the nature of their products, including whether they contain GM products or not. Any associated costs incurred to meet the requirements of importing nations are the direct burden of producers or their distributors.^70^

The Canadian government standardizes safety, testing, and labeling protocols for the good of all Canadian citizens, consumers, and producers alike. What is not apparent, however, is why there is not a greater acceptance by Canadian consumers of the well-researched and implemented protocols regarding the approval of GM food products. To understand this opposition to the voluntary labeling of GM food products, the nature of contemporary nongovernmental agencies (NGOs) must be understood.

### Nongovernmental Organizations (NGOs) and GM Labelling

Civil society organizations have played an important role in shaping public debate and media coverage surrounding agricultural biotechnology and food labeling policy in many jurisdictions.^25,71^ While several advocacy organizations have actively campaigned for mandatory GM labeling in Canada, the number of organizations directly involved remains relatively small compared with the broader Canadian population, and survey evidence suggests that public attitudes toward biotechnology remain heterogeneous.^26^

In Canada, as in many other countries, several NGOs have advocated for the introduction of mandatory labeling of GM foods on the basis that such a policy enhances consumer transparency and informed choice. However, from the beginning, Runge and Jackson^23^ found evidence that NGO-driven labels may create implicit negative signaling effects through bounded rationality. More recent research has examined the economic incentives facing advocacy organizations operating within information markets. Scholars have noted that advocacy organizations operate within competitive information environments in which public attention, funding, and organizational visibility are important resources.^72^

Research examining advocacy dynamics suggests that heightened public concern surrounding emerging technologies can increase engagement with advocacy campaigns and organizational support such as donations, media attention and organizational visibility.^73^ While these dynamics do not apply uniformly across all civil society organizations, they highlight the complex interactions between information dissemination, advocacy activity and public perceptions of biotechnology.^74^

These dynamics do not necessarily imply intentional misinformation; rather, they highlight how differing institutional incentives can shape the way complex scientific information is presented to the public. In the context of biotechnology policy debates, advocacy organizations, industry groups, and government institutions frequently present contrasting interpretations of scientific evidence and regulatory priorities.^75^ Understanding these interactions is important for evaluating policy debates surrounding GM food labeling. The policy process often reflects the interaction of scientific risk assessment, public perception, advocacy activity, and economic considerations, all of which contribute to the broader institutional landscape shaping biotechnology regulation^76^

Empirical evidence suggests that NGOs exert a significant influence on consumer behavior by shaping informational environments and perceptions regarding GMs, particularly through labeling advocacy. Reisch and Zhao^77^ argue that if increased GM labeling genuinely reflects public will and facilitates rational decision-making, it should elicit measurable shifts in consumer behavior. Adalja et al.^24^ counterbalance this claim by asserting that, in the absence of credible harm, enhanced information should not alter consumption patterns. A salient example of this dynamic is observed in Vermont’s legislative journey toward mandatory GM labeling, which culminated in the U.S. Federal Law (No. 764). Before implementation, the influence of information dissemination, shaped by framing and narrative,^78^ was evident through numerous valuation studies. Adalja et al.^24^ employed a quasi-natural experimental design to investigate consumer responses, utilizing a synthetic control constructed from neighboring Maine to isolate the legislative impact. Their analysis revealed that consumer awareness, as measured by Google Trends, preceded and predicted the increased adoption of non-GM products, accounting for 36% of new product uptake even before the law’s enactment. However, the formal introduction of mandatory labels did not induce further demand shifts, suggesting that the act of information dissemination, rather than the label itself, was the primary driver of behavior change. These findings support two central conclusions: first, that consumer behavior is strongly influenced by awareness-building campaigns, and second, that voluntary labeling might achieve disclosure goals more cost-effectively than mandatory schemes. Applied to the Canadian context, this implies that NGO-driven narratives and timing of awareness initiatives are critical in shaping market behavior, and that consumer choices are often predetermined, rendering post-label interactions less impactful. The widespread demand for mandatory labeling, therefore, might reflect the influence of a vocal minority rather than a fully informed public consensus.

The push for mandatory GM labeling, frequently attributed to the efforts of NGOs, has been characterized less as a response to consumer demand based on health or safety concerns and more as a campaign emphasizing the “right to know.” These NGOs have exerted a significant influence on consumer behavior by shaping public discourse, raising awareness, and encouraging regulatory change, even when scientific consensus affirms the safety of GMs.^21,24^. In doing so, NGOs manufacture demand for transparency, prompting producers to either disclose GM content or reformulate products to avoid labeling. The Vermont labeling case illustrates this dynamic; before label enforcement, heightened awareness and indirect effects of the legislative process shifted consumer preferences toward non-GM products, indicating that information campaigns, rather than labels themselves, predominantly influence behavior.^24^ This suggests that consumer responses are shaped less by post-label information processing and more by prior narratives promoted by NGOs, thereby revealing how these organizations function as agents of behavioral change through informational advocacy rather than scientific mediation.^77,78^

The regulatory demands driven by NGO advocacy, while politically salient, entail substantial economic trade-offs. As Bovay and Alston^21^ argue, if consumers exhibit indifference toward GM disclosure, then mandatory labeling might provide negligible benefits while imposing significant costs. Hence, NGO influence on consumer behavior extends beyond awareness to shape market dynamics and regulatory structures, often with unintended economic consequences for both producers and consumers.

### Consumer Attitudes Toward GM Foods and Canadian Consumer Labeling Demand

A substantial body of international research has examined consumer perceptions, acceptance, and willingness to pay for GM foods (e,g.,^73,75,76,78–83^). Experimental and survey-based studies have explored how consumers respond to different types of biotechnology information and labeling systems. For example, research by Boccia and Punzo^83^ demonstrates that consumer acceptance of genetically modified foods varies depending on the perceived benefits of the technology and the level of information provided to consumers. Similarly, studies examining willingness to pay for biotechnology-related food attributes highlight the importance of labeling design and information framing in shaping consumer responses. Work by Waterfield, et al.^82^ also shows that consumer preferences expressed in surveys might differ from voting behavior or actual purchasing decisions, illustrating the complexity of consumer responses to biotechnology labeling policies.

Additional studies emphasize the role of knowledge, risk perception, and trust in shaping consumer attitudes toward GM foods. Research across multiple countries has found that consumers’ willingness to purchase biotechnology-derived foods is strongly influenced by perceptions of health risks, and trust in regulatory institutions.^79,81^. Social media dynamics and misinformation can also affect consumer perceptions, as demonstrated in studies examining how online communication and expert messaging influence attitudes toward biotechnology.^75^

There have been attempts, both governmental and academic, to understand the relationship Canadian consumers have with GM foods. In a report submitted to and adopted by Health Canada, The Strategic Counsel indicates that “Canadian consumers’ understanding and impressions of GM foods could be described as not that well-formed, as demonstrated by the lack of detailed knowledge.”(^84^, p. 4^4^4“Not that well-formed … ” in the quote from the Strategic Counsel refers to the opinions held by the surveyed public seem to be based on a low understanding of food science and technology. The low level of scientific literacy extends to agricultural practices, market implications of technology, and quantitative consequences of consumer preferences.Negative or conflicting views can be attributed to messaging from anti-GM advocates and environmental groups.^84^ The report states that negatively biased media coverage, in conjunction with NGO anti-GM activities, has largely shaped public opinion. The report noted that up to 78% of consumers did not believe that the voluntary labeling scheme employed in Canada was sufficient or credible and would be in favor of a mandatory labeling requirement. Furthermore, if such a program were instituted, the increased transparency and enhanced ability to make informed decisions would likely result in 62% of surveyed consumers actively avoiding GM-labeled food.^(84, p. 39)^ However, the same report found that only 7% of respondents indicated that they frequently look for information about GM content on food labels, suggesting a wide gap between expressed preferences for labeling and actual consumer behavior.^84^ The findings reveal a significant disparity between scientific communication and consumer acceptance of scientific facts.

Other studies conducted by Baynham^85^ and Charlebois, et al.^31^ found contradictory evidence regarding Canadian consumers’ attitudes to mandatory GM food labeling. While many Canadian participants in these studies believed GM foods to be safe, many also were uncertain about their health effects. While a high proportion of participants would passively support mandatory GM content labeling, these same participants indicated that product price was almost three times more important than knowing the item contained GM ingredients.

Survey responses regarding food labeling can be influenced by question framing. The influence of new media platforms and content creators, that seek to maintain engagement through sensationalism over content, do so by reframing consumer relationships to GMs. Ryan et al.^72^ identify independent agents, absence of government oversight, being able to monetize the persistent uncertainty around GM products despite the lack of evidence of unsafe consumption. Questions that explicitly reference a particular attribute – such as genetically modified ingredients–might prompt higher levels of expressed support than more neutral survey questions. Studies using alternative approaches, such as asking respondents to identify missing information from hypothetical food labels, often produce much lower responses for GM-related information. For example, research summarized by the International Food Information Council found that when consumers were asked to identify information missing from labels without prompting specific attributes, only about 2% of respondents mentioned GM ingredients.^86^ This distinction reflects a broader methodological difference between stated preferences expressed in surveys and revealed preferences reflected in actual consumer behavior.

The mixed response to the desire for mandatory labeling by Canadian consumers, even when uniformly informed about GM foods, presents a distinct problem for the Canadian government. While several advocacy organizations have actively campaigned for mandatory GM labeling in Canada, the number of organizations directly involved remains relatively small compared with the broader Canadian population, and survey evidence suggests that public attitudes toward biotechnology remain heterogeneous. Even if the current voluntary labeling system has been deemed sufficient by the Office of Consumer Affairs (OCA) at Health Canada and the CFIA, there is evidence of the public’s general confusion and mistrust of GM foods. New legislation, including revised labeling schemes and educational initiatives, would need to address any underinformed biases held by consumers.

## Assessing the Potential Costs and Benefits of Mandatory Labeling

Responding to consumers’ and NGOs’ demands for GM labeling would entail extra costs and changes in consumer demand. Producers might also react by reformulating products to avoid labeling them as containing GM ingredients, replacing those ingredients with non-GM ingredients produced using conventional technology or organic methods. This indicates that both information asymmetry and additional production pressure can negatively affect consumer WTP and create a negative demand shock when labeling is mandated.^80^ Ultimately, these additional costs would become a burden on consumers or result in a reduction in net margin among supply chain participants.

Bovay and Alston^22^ concluded that “PL 114–216 is worse than a complete absence of mandatory labelling laws … is a clumsy and incoherent piece of legislation that will be burdensome initially to producers and ultimately to consumers–potentially, to the tune of billions of dollars in annual expenses, if the competitive response of marketers to the law is a substantial increase in demand for non-GM inputs and products, an outcome that may result even if (most) consumers express indifference toward the disclosure statements” (p.14, 24). They argued in a 2016 paper that if consumers and marketers are indifferent toward information about GM content or the disclosure statements regarding specific products, the best strategy might be to divulge content preemptively but not to change anything else about operations or marketing.^21^ Increased labeling standards, applied domestically or internationally, might only compound the cost burden for producers and governments that implement the change. It is then incumbent upon a legislative body to carefully assess and analyze the cost–benefit ratio before acting, either by increasing labeling requirements or by other means, to satisfy consumer demand.

### Potential Mandatory Labeling Costs

As the U.S. is Canada’s largest trading partner and shares similarities with Canada both culturally and legally, the Bovay and Alston^22^ study provides a template for estimating many of the costs that might be faced in Canada if mandatory labeling of GM products was to be implemented. A comprehensive summary of their findings, in conjunction with that of other contributors, follows and is further illustrated in Table 1.

**Table 1.** Estimates of compliance with mandatory GM labeling laws.

Type of cost	Estimated annual cost: USD	Source	Context of estimate	Scaled to US 2016 population (323 m)*	Scaled to Canada 2023 population (40.9 m), CAD, adjusted for proportional value ([k ×Pv]/ratio)**
Labeling	$1104/per product (one time cost)	Shepherd-Bailey^17^	California Prop. 37	n/a	n/a
Labeling	$3.8b (one-time cost/ consumer) $2.3 b (one-time cost/ manufacturer)	Dunham^20^	Vermont Act 120 response	$380 m/year for 10 years $230 m/year for 10 years	$56.5 m/year for 10 years $34.2 m/year for 10 years
Complete Replacement: GM->non-GM inputs	$11–103/capita	Lesser and Lynch^19^	New York Act 617	$3.6–33b/year	$12.9–121/capita; $525 m–4.9b/year
Complete Replacement: GM->certified organic inputs	$90–388/capita	Lesser and Lynch^19^	New York Act 617	$29–125b/year	$105.8- 456.2/capita; $4.3–18.6b/year
Segregation, Certification, and Monitoring	$1.2b	Alston and Sumner^16^	California Prop. 37	$11.6b/year	$1.73b/year
Segregation, Certification, and Monitoring	$9/capita	Lesser and Lynch^19^	New York Act 617	$2.9b/year	$432 m/year
Warehousing and Retail Space	$39-45 m	Lesser and Lynch^19^	New York Act 617	$640–740 m/year	$95.25–110.1 m/year
Total Min. Cost of labeling, alone^a^				**Up to $610 m/ year for 10 years**	**Up to $90.7 m/year for 10 years**
of Remaining Cost Categories^b^				**$7.1–137b/year**	**$1.1– 20.4b/year**

Source: Bovay and Alston^22,87,88^; Bank of Canada^89^; Authors’ Scaled Calculation 2024.

Estimates of costs associated with mandatory labeling laws in the U.S. because of changes to the national bioengineered food disclosure standard (NBFDS).

Canadian scalability in 2023 values.

Calculation value identifiers: **k** = calculated costs in USD in 2012–2016; **Pv** (proportional value)=A × Var. = 1.243 × 0.9459 = 1.1758 (A = compounded rate of inflation, calculated annually for 10 year at an average annual rate of inflation for Canada of 2.2% (similar to US average annual rate of inflation of 2.1%)and Var.=variance in exchange rate between USD and CAD between January 2014 and December 2023 of +5.7% CAD); **ratio** = average historical US population January 2014/current Canadian population December 2023.

^a^Approximate minimum cost of changing labeling alone would amount to CAD 0.84 annually per product per person for up to 10 years.

^b^Approximate remaining costs of replacement, segregation, certification, monitoring, warehousing, and retail space would amount to CAD 262.8306 annually per person. Calculated at median value of Total Cost of Remaining Cost Categories/Canada Population 2023 (CAD 10.75b/40.9 m).

^c^All values attributable to Bovary and Alston,^22^ and in conjunction with source data associated with the article, and^87,88^); Bank of Canada.^89^

^c^Labeling costs include figures for both consumers and manufacturers as per the approximate values found in Dunham.^20^

Shepherd-Bailey^17^ analyzed three potential costs associated with Proposition 37, the California Right to Know Genetically Engineered Food Act: relabeling expenses, litigation-related costs, and additional administrative costs. Based on FDA estimates, the one-time expense to redesign labels was found to be USD 1,104.43 per product, representing 0.03% of the average annual per-product sales. A secondary cost of store placards disclosing GM status would be USD 2,820 per store, representing 0.01% of the average supermarket’s sales.^90^

Dunham^20^ focused on the potential cost impacts of Vermont’s GM labeling law. While manufacturers might simply label their products in accordance with the new GM labeling mandates, the potential cost that Dunham^20^ studied is the shift of manufacturers using a mix of GM and non-GM ingredients to *only* non-GM ingredients. The shift is postulated as being the result of manufacturers seeing a market disadvantage to being associated with GM products. The study evaluated three separate costs across a range of 41 different food and beverage categories in a straightforward input-output accounting model. The results showed a one-time cost to manufacturers of approximately USD 3.8 billion, and a one-time cost of approximately USD 2.3 billion due to a regulatory change that mandated GM labeling. As distributors and/or retailers would likely not shoulder the USD 6.1 billion cost for facilitating a product’s commercial path, these costs would likely be passed on to the consumer to ensure a producer’s normal profit. Whether the extra expense can be seen as a “right to know” tax or the price associated with an enhancement of the range of available consumer choices was beyond the scope of the researchers to determine.

Lesser and Lynch^19^ investigated two specific areas of recurring costs associated with changes in legislation and GM labeling: replacement and segregation. The Lesser and Lynch^19^ study utilized data on consumption trends from New York State, assuming that all cost increases would ultimately be passed on to consumers. The study calculated that 40% of all items in the New York supermarket environment would be subject to a change in mandated food labeling, which ranges from 21,000 to 25,000 separate items and represents 50–58% of all items available in supermarkets. In a hypothetical market environment, if no GM products were deemed acceptable to consumers, replacements for those foodstuffs would have to be found. Lesser and Lynch^19^ proposed two approaches to compliance, using either non-GM or organic inputs as possible options. Replacing GM ingredients with non-GM ingredients would likely result in higher ingredient costs and increase prices by USD 11 per capita annually, or approximately USD 3.6 billion annually when scaled to the American population. Replacing GM ingredients with organic ingredients likely would result in higher ingredient costs and increase prices by USD 90 per capita annually. The extra costs related to segregation, keeping non-GM ingredients and food products separated from those that contain GMs also entailed extra costs. From basic inputs to finished products on the shelves, GM foods would need to have a verifiable and accountable logistics chain. This segregation would require additional warehousing, retail, manpower, and operating space costing an average USD 2 per capita annually. Additional manpower would also be needed to enable documentation, identity preservation of both non-GM and GM products, enforcement agencies that check and maintain segregation standards, and agencies that design, regulate, and verify the final labeling standard. These ongoing costs would average USD 9 per capita annually.

Alston and Sumner^16^ studied the economic implications for farmers and the food industry regarding Proposition 37, the California Food Labelling Initiative. They found that little or no benefit would be derived from a costly regulation shift to a mandatory GM labeling scheme. Not only would Proposition 37 impose greater costs on consumers directly, but the initiative would also affect others in the food supply chain, with farmers experiencing the greatest impact. They found that USD 1.2 billion would be needed to meet segregation, certification, and monitoring requirements for California alone, or USD 11.6 billion annually when scaled to the American population.

Table 1 includes a calculation for proportional Canadian scalability in 2023 Canadian dollar values, with all values sourced from^87,88^; Bank of Canada.^89^ Estimates of costs if Canada were to require mandatory labeling of GM products (based on the various studies summarized above, adjusted to Canada’s 2024 population, the relative value of the Canadian dollar, and inflation experienced since the time of the above studies) are summarized in Table 1.^5^5The formula used was: ([k ×Pv]/ratio) where: k equals scaled calculated costs in USD in 2012–2016; Pv is the proportional value derived from A × Var. (where A is the compounded rate of inflation, calculated annually for 10 years at an average annual rate of inflation for Canada of 2.2% (similar to US average annual rate of inflation of 2.1%) and Var. is the variance in exchange rate between USD and CAD between January 2014 and December 2023 of +5.7% CAD); and ratio is the average historical U.S. population on January 2014 compared to the current Canadian population in December 2023.Based on the estimated cost of approximately CAD 265 per person annually, the cost for an average Canadian household of four would be approximately CAD 1,060 per year. According to the 2026 Canadian Food Price Report, the average household of four is expected to spend approximately CAD 17,500 on food annually. Thus, mandatory GM labeling could increase household food expenditures by roughly 6% relative to projected food spending.

As the overall collation of data in Table 1 shows, physical labels, while not insignificant in cost, are significantly less costly compared to the cost of maintaining regulatory support and facilitating increased consumer awareness. Table 1 illustrates the extra costs (or remaining costs) of replacement, segregation, certification, monitoring, warehousing, and retail space associated with implementing a mandatory GM labeling scheme. For labeling alone (or the minimum cost of changing labeling alone), the U.S. would likely incur a cost of more than USD 6.1 billion, and the Canadian equivalent would be CAD 907 million. For all other costs associated with a change in labeling scheme, it is estimated that the US would incur more than a minimum cost of USD 7.1 billion annually, and the Canadian equivalent would be a minimum cost of CAD 1.1 billion annually.

These costs, conservatively estimated, appear disproportionate to the benefit received, if, as noted previously, price is the primary factor influencing consumer behavior. Combined, if the analysis of the U.S. market and legislative cultures remain comparable to Canadian market and legislative cultures, the combined cost for Canadian consumers would be more than CAD 265 per person annually.^6^6“Combined cost” refers to approximate remaining costs of replacement, segregation, certification, monitoring, warehousing and retail space would amount to CAD 262.83 annually per person [calculated at median value of Total Cost of Remaining Cost Categories/Canada Population 2023 ($10.75b/40.9 m)] PLUS Total Minimum Cost of Labeling of CAD 2.22 annually per person [calculated by Total Minimum Cost of Labeling, Alone/ Canada Population 2023 ($90.7 m/40.9 m)].

### Potential Benefits of Mandatory Labeling

#### Trade: Existing Canadian Canola Sector Relations

Currently, canola’s success within the Canadian agri-food sector is largely attributed to its ability to supply the global market through significant exports, despite its limited domestic market. As Canada is the world’s largest producer of canola, this remains a significant economic advantage for the Canadian economy. Canada produces approximately 20 million tonnes of canola annually, with roughly half of this amount exported.^34^ The U.S. has been Canada’s largest export market, accounting for more than 85% of all canola meal and up to 60% of total canola oil exported. Canola meal is an important feed ingredient for poultry, swine and dairy cows.^1^ The U.S. market has accounted for CAD 8.6 billion in export sales.^1^

Since the full implementation of mandatory labeling of GM food products in the U.S. on January 1, 2022, there has been very little impact on Canada’s trade in canola with that country. Canola oil is stripped of its DNA during the refining process for food use; therefore, Canadian-produced GM canola does not require mandatory disclosure.^91^ The USDA *does* require bioengineered ingredients to be disclosed if the genetic material is detectable. The U.S. regulation states that “Food from an animal cannot be declared bioengineered on the basis that the animal has been fed bioengineered food.”^92^ This is good news for Canadian canola growers who sell their refined product in the U.S.

According to the Canola Council of Canada,^1^ secondary markets account for what remains of the 10.5 million tonnes exported worldwide. China, Mexico, Japan, and the United Arab Emirates (UAE) historically have been the largest importers of Canadian canola. In 2023, China imported CAD 5 billion, Mexico imported CAD 1 billion, Japan imported CAD 883 million, and the UAE imported CAD 196 million of Canadian canola and canola products. These countries have their own safety and regulatory guidelines surrounding GM products. However, there is little evidence that labeling and other regulatory requirements in these major markets for Canadian canola contributed to any diminishing of trade with Canada.

Mexico is a member of the Canada–United States–Mexico Agreement (CUSMA), and until 2023, was a large importer of Canadian canola. On April 17, 2024, Mexico enacted the “General Law on Adequate and Sustainable Food” (LGAAS, by its Spanish acronym). The new law has two primary objectives: (1) mandate warning label usage for products containing genetically engineered ingredients and (2) incorporate socio-economic considerations into Mexico’s national food policy.^93^ The aim of LGAAS is to establish a new and comprehensive level of food sovereignty focusing on self-production and “rights-based legislative framework for achieving healthy and sustainable nutrition for all.”^94^ Of relevance to the Canadian canola sector are *Articles 5* and *21*, in which:

*Article 5* obligates State authorities to promote, respect, and protect the exercise of the right to adequate food through precautionary measures. Article 5 utilizes the “precautionary principle” in relation to GM food products and ingredients, allowing for the unilateral restriction of foods that lack the “burden of proof.”^7^7The precautionary principle is decision making paradigm with four central components: engaging in preventive action in the face of “uncertainty”; allocating the “burden of proof” to the proponents of a possibly harmful activity; creating alternatives to possibly harmful actions; and increasing public participation in decision making.^113^

*Article 21* involves mandatory warning label requirements for products that contain GM ingredients: “Producers and distributors of processed foods must warn, in addition to the elements required in article 212 of the General Health Law, if its products contain ingredients that directly derive from using genetically modified organisms in terms of the Law.” Article 21 does not specify to which law “ … in terms of the Law” refers,”^93^ (p. 3). *Article 21* mandates standardization and infrastructure to implement and manage the new labeling scheme. Development of such structures and tools would necessitate an indeterminate time frame in which the importation or use of Canadian canola would likely be reduced.

Since Mexico has been a significant importer of Canada’s GM canola, the ramifications of this new legislation are already apparent. Prior to 2023 (2014–2022 inclusive), Mexico imported approximately 14.05% seed, 3.21% canola oil, and a nominal .36% canola meal from Canada. However, canola seed and canola oil imports fell by 50% and 30%, respectively, in 2024, with no canola meal imports reported.^1^ While the present export value to Mexico is approximately CAD 1.0 billion, the 31.4% increase in market value of canola (December 2014 to December 2024 inclusive) represents an over CAD 1.2 billion loss in trade.^95^

During the development of China’s regulations on mandatory labeling of GM food products, heterogeneous responses to labeling information were observed. Enhanced information regarding GM food products had stronger effects on consumers initially unwilling to buy GM foods, suggesting that label design can shift demand where uncertainty is high.^96^ Despite the mixed consumer response, imports of Canadian canola increased.^1^ While there is some protectionism surrounding Chinese domestically developed and grown GM crops, the short fall in their own raw and refined products continues to be imported from Canada.^97^

Another example of different regulatory practices surrounding GM products not inhibiting strong trade relationships between nations is that of Japan and Canada. Japan, a country with some of the strictest regulations involving GM importation, allows the importation of Canadian canola and its refined products. Early in 2023, the Japanese Consumer Affairs Agency (CAA) updated their agricultural product list that requires mandatory GM labeling, except for nine major crops (one of which is canola) that have been allowed to be sold in Japan as it currently does not grow any of its own.^98^

#### Trade: Creating New Canadian Canola Sector Relations

Canada’s greatest opportunity for expanding its international trade lies with countries with which it currently has little or no trade. Underdeveloped international relations are an understandable barrier to such development. The probable, yet unfounded, bias against GM products and the inability to overcome this intolerance are other likely issues that inhibit the formation of new trade relationships.

The largest roadblock to increased exports of Canada’s GM canola is unfounded bias and prejudice against GM products. According to Kalaitzandonakes and Bijman,^99^ the EU’s labeling policy, established in 1997, set the precedent for the first broad rejection of GM products by legislators and retailers. The EU’s rejection of GM products was founded on an extreme version of the precautionary principle. The EU’s implemented mandatory labeling regimes, that require disclosure when GM ingredients are present above specified thresholds, is internally inconsistent because it restricts domestic cultivation while allowing extensive GM imports and consumption through feed and supply chains. European regulation is process-based, rather than product-risk-based, creating regulatory incoherence and potential opportunity costs for agricultural innovation.^100^

As a result, the legislators continue to require GM foods to be labeled. In response to the new GM labels, EU retailers assumed that these products would be unwelcome to many of their consumers. As retailers in the perishable foods industry typically do not want to carry inventory that experiences little or no turnover, the default position was *not* to acquire products manufactured or labeled with GM content. Hence, the rejection was not by consumer choice but by an absence of choice. This suggests that the EU policy was not designed to maximize strategic trade.^101^

Smyth^70^ hypothesizes that the single biggest regulatory trading barrier to GM products is the misinterpretation and application of the Cartagena Protocol on Biosafety (CPB), ratified in 2003^102^. The CPB is an EU initiated United Nations negotiated agreement ostensibly in reaction to the WTO’s unwillingness to reopen the Sanitary and Phytosanitary (SPS) Agreement, an earlier agreement setting out the basic rules for food safety and plant and animal health standards and allows countries to set their own standards achieved and ratified in 1995. While both the SPS and CPB stress a nation’s sovereignty in creating its own protocols relating to food safety, the CPB makes specific requirements about GM crops and foods. As the WTO requires standards to be based on verifiable science, the lack of evidence supporting the hazards of GMs did not induce the WTO to reassess the SPS.^103^

As a result of the CPB, 65 countries now have mandatory labeling laws around GM concerns. Most of these countries have some reasonable tolerance for the presence of GMs, exemplified by the 0.9% by weight limit for any individual ingredient in the EU^18^ or the 5% by weight limit of the finished product for processed foods that contain certain ingredients in Japan.^104^ The nuance of this type of legislation is often lost on developing nations, and thus their participation with the CPB seems more politically and trade-driven, as opposed to ensuring the safety and sovereignty of their nation.^22,104,105^

While leading GM crop-producing countries such as Argentina, Australia, Canada, and the U.S. have not ratified the CPB, 170 countries *have* signed on to the agreement. This might indicate the level of influence and trading power that the EU exerts. Through direct diplomacy and the use of NGOs to inhibit the adoption of GM crops and food products, the EU seems to be in direct violation of the WTO ratified food sovereignty as outlined in the SPS. The effect is that trade with nations that have ratified the CPB is stifled, leaving both developing countries and GM producers with a net economic loss of over USD 10 billion annually.^70,^^8^8Countries growing GM crops are: Argentina, Australia, Bangladesh, Bolivia, Brazil, Canada, Chile, China, Colombia, Costa Rica, Czech Republic, Honduras, India (Bt cotton only), Malawi, Mexico, Myanmar, Nigeria, Pakistan, Paraguay, Philippines, Portugal, South Africa, Slovakia, Spain, Sudan, eSwatini (Swaziland), U.S., Uruguay, Vietnam, and Zambia.^115^

With only 15% of the world’s nations open to producing GM crops (30 nations out of 195), it seems exceedingly unlikely that Canada will acquire sufficient new trading partners to bring any noticeable benefit from trading GM products. Hence, in the short run, maintaining canola yields and quality must take priority as Canadian producers focus on meeting existing trade agreements and increasing national consumption.

## Overall Assessment

A deeper understanding of Canadian canola development and modern production practices has indicated that GM canola production poses no harm to the environment or consumers (e.g.,^30,33^). The techniques used to produce GM canola have increased yields, improved drought tolerance, and reduced the overall use of industrial fertilizers, herbicides, and pesticides (e.g.,^106,114^). Through a cleaner environment and a lack of GM attributable health or safety issues, Canadian canola farmers can be confident in their product.

In conjunction with producers, the Canadian government has shown initiative in developing and maintaining its GM voluntary labeling scheme, which has demonstrated to be cost-effective, scientifically rigorous, and reliable (e.g.,^107,108^). The Canadian government has continued to ensure the highest possible standards with regulatory practices (e.g.,^65^). In contrast to Canada’s voluntary labeling scheme, mandatory GM labeling schemes are currently in place in 65 countries, including major export destinations for Canadian products such as the U.S., Mexico, China, and Japan. However, to date, there have been little or no negative long-term trade consequences overall with the alignment of Canada’s labeling system with the mandatory labeling systems of these major importers of Canadian canola and canola products. As Canada’s production and yield of canola have increased, so has its trade (e.g.,^1,39^).

The U.S., Canada’s closest and largest trading partner, is also culturally, legally, and ethnically similar to Canada. The U.S. has recently instituted a federal mandatory GM labeling scheme in answer to both NGOs influence and US consumer concerns. Solid U.S.-based research has already demonstrated that increased legislation is likely to have a negative impact on the market by increasing costs and providing false-positive confirmation of the threat posed by GM products (e.g.,^22^). It was also shown that the cost of implementing a mandatory labeling requirement would be significant in Canada too.

While there has been a general trend for nations to have more restrictive labeling standards, these initiatives have been shown to be driven largely by NGOs and related consumer advocacy groups (e.g.,^25,109,110^). The impetus behind these campaigns has, according to the NGOs themselves, been to protect the world’s consumers from the possible dangers of GM consumption and argue that the “right to know” if products contain GM content is a basic right (e.g.,^27^). To clarify, the WTO and WHO have stated that there is no scientific evidence indicating current or potential dangers from approved GM products.^14^ There have, however, been quantifiable negative results attributable to NGO influence, as they have effectively undermined citizens’ confidence in the scientific method and governments’ accountability (e.g.,^74^).

The assessment of possible additional trade benefits with a decreasing emerging market hints at possible challenges ahead for the Canadian government and Canadian canola producers (e.g.,^70^). With the advent of the CPB, more countries are choosing more restrictive GM trade and labeling processes. With increased regulation, barriers to trade can be expected (e.g.,^70^). Increased gains due to new international trade are apt to be offset by the increased costs associated with any new or mandatory regulatory compliance (e.g.,^22^). Therefore, a concerted effort to develop domestic market acceptance of GM products is a key step in securing the continued presence of GM food and health biotechnology companies in the Canadian agriculture sector.

## Conclusion and Policy Recommendations

Canola is a dominant crop in Canada with firmly established recognition and market presence on an international level. Most of the canola grown in Canada nowadays is from genetically modified stock, resulting in its classification as a genetically modified (GM) product. Many countries, including recently the U.S., require mandatory labeling of GM products, which exceeds Canada’s voluntary labeling scheme. The U.S. has been Canada’s largest export market, accounting for 85% of canola meal and 60% of canola oil exports. The implications of these changes for the Canadian canola industry and economy are not well understood.

This paper assessed the justification and potential effects of replacing Canada’s voluntary GM labeling scheme with a mandatory GM labeling scheme for the Canadian canola industry and economy. In particular, the paper examined the economic consequences, i.e., the potential benefits and costs associated with mandatory labeling of GM foods in Canada for the Canadian canola industry.

The findings show that Canada’s canola sector continues to operate with a high level of efficiency, meeting current domestic production and international trade requirements that are minimally affected by labeling regulations. Furthermore, Canada’s voluntary labeling scheme has not hindered exports of canola oil or meal exports despite mandatory labeling requirements for GM food products in major export markets. It was also shown that the cost of implementing a mandatory labeling requirement for GM food products in Canada would be significant, which could potentially have a negative impact on the canola sector and the economy. Bovay and Alson^22^ also demonstrated that the recent change to the mandatory GM labeling regulations in the U.S. resulted in little to no economic benefit, but likely a substantial ongoing cost. Given that food price inflation in Canada has recently exceeded that of most G7 countries and that food affordability is a major concern for many households, policymakers considering mandatory labeling policies should carefully weigh the informational benefits of such policies against their long-term cost implications for consumers.

Canada faces domestic pressure regarding the labeling of GM products, as a large proportion of Canadian consumers express a desire for more information. This “right to know” demand is largely created by nongovernmental organizations (NGOs) and other consumer advocacy groups, rather than the scientific or agricultural communities, and influences the public.

With a better understanding of the economic, social, and regulatory issues surrounding the GM labeling regulations, several policy recommendations could be made that would contribute to the canola sector’s growth, potential improvements in individual wellbeing, and an increase in overall social welfare. Initially, it is recommended that the Canadian government adopt a “static,” yet “active,” position regarding the existing Canadian labeling system. An “active static” position can be seen as retaining the current voluntary labeling regulations but remaining open to re-assessing labeling requirements on a regular basis with the input of private sector representatives. The voluntary labeling scheme, as it exists in its present form, has proven sufficient to ensure the safety of GM food products. Since there is little evidence to suggest that these GM food products warrant special attention, the existing system is sufficient. However, it is incumbent for the agencies within the government that create and monitor such policies to remain actively vigilant to new information and concerns about GM foods and food labeling.

As noted previously, many Canadians continue to view approved GM foods as potentially harmful^84^. Regardless of the causes of this misinformed belief, citizen and consumer confidence are key elements in a smoothly working market economy. A reasonable way to combat erroneous beliefs is education. Supporting the voluntary labeling scheme with increased and sustained public education on the safety of GM food products will expand consumer awareness. Consumer confidence in the Canadian labeling regulation process can be improved by making the process transparent at the provincial and federal levels. Lastly, educating Canadian citizens and consumers about the structure, role, and influence of NGOs might mitigate the extent of power these organizations hold over government regulations and public opinion.

One final recommendation would be for the Canadian government to regularly schedule reassessments of its voluntary GM labeling system. The inclusion of representative consumer bodies in this assessment process might enhance consumer confidence in both the regulatory system and the safety of GM products. By regularly examining the continued viability of its systems, the Canadian government will be sufficiently aware of any specific domestic or international concerns that might warrant active changes. Changing a system when it is unnecessary is illogical and costly. Failing to change a system when it is critically necessary is irresponsible and dangerous.

To sum up, the Canadian GM canola sector has been highly successful both domestically and internationally. EASAC^111^ concluded: “There is no validated evidence that GM crops have greater adverse impact on health and the environment than any other technology used in plant breeding. There is compelling evidence that GM crops can contribute to sustainable development goals with benefits to farmers, consumers, the environment, and the economy” (p. 2). GM foods present great potential for dealing with the recent challenges, such as a need for products with high yield/production, reduced environmental footprints, and enhanced health benefits. However, international market access is important for further success and growth in the Canadian GM canola sector. While the Canadian canola sector has been a successful exporter, a significant challenge to further growth in global markets is the presence of international non-tariff trade barriers.

The issue of increased GM labeling requirements could pose a significant obstacle to the further growth and development of the Canadian canola sector with significant adverse implications for Canadian consumers and the Canadian economy. Awareness of this crucial and timely issue, along with regular reevaluation of current developments, is essential and vital for the Canadian canola sector and the economy as a whole.

Increasing the impetus for evidence-based policy development is vital. Increasing consumers’ awareness of the benefits of GM crops, their contribution to human health, and the effects on environmental sustainability are also crucial issues. Lastly, it is essential that the appropriate governance framework includes stakeholders and members of the public to ensure that policies are based on a shared vision of the future.
